# Behçet's Syndrome in a Child: A Case Report and a Review of Literature

**DOI:** 10.7759/cureus.67273

**Published:** 2024-08-20

**Authors:** Muzun H Alatyan, Hind S Albahouth, Yara S Al Fahhad, Lama A Alotaibi, Ebtihal S Almutairi, Hadeel A Almalky, Asmaa A Faden

**Affiliations:** 1 Dentistry, King Saud University, Riyadh, SAU

**Keywords:** behçet's syndrome in children, colchicine, hla-b51/b5, joint pain, recurrent oral ulcers

## Abstract

Behçet's syndrome is a complex chronic inflammatory disorder characterized by widespread inflammation of the blood vessels, affecting various systems in the body. Although its exact cause remains unknown, genetic predisposition, particularly HLA-B51/B5 gene carriage, and environmental factors are believed to play roles. The disease typically manifests in individuals aged 20-40 years, with an uncommon occurrence in children and elderly individuals. Key clinical manifestations include recurrent oral and genital ulcers, skin lesions, ocular involvement, positive pathergy test results, and other systemic symptoms. Eye involvement is common and can lead to severe visual impairment if left untreated. This diversity of Behçet's disease (BD) presentations and complications emphasizes the importance of early recognition and management.

An eight-year-old girl presented with a deep painful ulcer in the mouth and a history of chronic constipation, severe joint pain, and recurrent mouth ulcers. Initial examination revealed an ulcer scar on the tongue and a deep ulcer on the left side of the mucobuccal fold. The patient was diagnosed with a recurrent major aphthous ulcer and prescribed Predo pediatric syrup as a mouthwash and paracetamol to relieve the pain. A biopsy was recommended by her physician to be done under general anesthesia and to rule out malignancy; the biopsy result revealed the presence of a benign squamous epithelium with reactive changes. The genetic result revealed HLA B*51 positivity and normal immunoglobulin levels. Treatment with colchicine led to the complete healing of the ulcer with scar formation after three months.

This case report highlights the unique presentation of Behçet's syndrome in children and the challenges associated with its diagnosis. It emphasizes the importance of the early recognition and prompt management of BD in the pediatric population, in which disease progression can be more severe than in adult-onset cases. This case provides valuable insights into the clinical features of and diagnostic approach to Behçet's syndrome in children.

## Introduction

Behçet's syndrome is a multisystem chronic disorder characterized by the inflammation of large and small blood vessels that affects various parts of the body, such as the skin, mucosa, joints, eyes, arteries, veins, nervous system, and gastrointestinal system [1,2]. The etiology of Behçet’s syndrome remains unknown, but genetic and environmental factors are believed to be involved. The HLA-B51/B5 gene, found in Japanese, Middle Eastern, and Turkish populations, is a common risk factor for the disease. Other genes, such as those encoding tumor necrosis factor (TNF) and heat shock proteins, may also play roles. Exposure to certain infectious agents may trigger an inflammatory response in genetically susceptible individuals, but the specific agents responsible have not been definitively identified [3].

Behçet's disease (BD) typically occurs in individuals aged 20-40 years, but can develop at any age, with uncommon occurrence in children and elderly individuals. The symptoms of BD in children may differ from those in adults, and the disease's progression is often more severe in pediatric cases, leading to delayed diagnosis [4].

The criteria for the classification of Behçet's syndrome were established in 1990 by the International Study Group (ISG) for Behçet's Disease [5] and updated in 2014 by the International Team for the Revision of the International Criteria for Behçet's Disease (ITR-ICBD) [5]. According to the 2014 ICBD, a patient with recurrent oral ulcers, genital ulcers, skin lesions, ocular involvement, positive pathergy test results, and other syndrome-related organ symptoms can be classified as having Behçet's syndrome [6]. The clinical presentation of BD is diverse, reflecting the varied patterns of vascular and tissue involvement [7]. Mucocutaneous lesions, such as oral and genital ulcers, are the hallmarks of BD, typically preceding the development of other systemic manifestations, the oral ulcers resemble benign, recurrent aphthous stomatitis and can range in size from minor to major to herpetiform ulcers, potentially leading to scarring [7,8]. Genital ulcers can occur in any part of the genitourinary tract and have the potential to cause scarring [8]. The various cutaneous manifestations of BD include erythema nodosum-like lesions, superficial thrombophlebitis, acneiform or pseudofolliculitis lesions, pyoderma gangrenosum-like lesions, pustular vasculitic lesions, cutaneous small-vessel vasculitis, and sweet syndrome-like lesions [8]. BD can also present with ulceration in various parts of the gastrointestinal tract. These ulcers may be similar to those found in inflammatory bowel disease [8]. Fifty percent of patients with BD have musculoskeletal symptoms, such as arthritis or arthralgia which can substantially impact patients’ quality of life [2,7]. Neuro-BD is a severe complication that occurs in approximately 10% of BD cases [2]. Thrombophlebitis, in both superficial and deep forms, is the most frequently observed vascular manifestation of BD in the lower extremities and can result in life-threatening complications [2,7]. Eye involvement is common in BD, affecting more than half of patients, particularly young males, and can lead to severe visual impairment if untreated. Eye symptoms typically occur within the first few years after BD diagnosis and are rare in later stages when not present earlier [2,8]. Laboratory findings with BD are typically nonspecific. The erythrocyte sedimentation rate (ESR) and C-reactive protein (CRP) level may be mildly elevated, particularly in cases featuring arthritis, erythema nodosum-like lesions, or vascular issues, but no autoantibody such as rheumatoid factor or antinuclear, anticardiolipin, or antineutrophil cytoplasmic antibody (ANCA) is typically detected. Levels of anti-*Saccharomyces cerevisiae* antibodies may be higher in patients with BD and gastrointestinal involvement than in those without such involvement [9]. BD also lacks specific histopathological features. The most significant genetic association with BD, involving the HLA-B51 gene, was initially documented by Ohno et al. [10]. Human leukocyte antigens-B51 (HLA-B51) positivity rates among individuals with BD from diverse ethnic groups range from approximately 40% to 60% [11]. Nevertheless, the diagnostic utility of HLA-B51 in routine clinical practice is limited due to its high prevalence in the general populations of countries where the prevalence of BD is high [12].

The 1990 ISG criteria are used most commonly for the diagnosis of BD [5]. They were developed by a panel of experts who closely monitored a large group of patients with BD in daily clinical settings. The presence of oral ulcers is mandatory for the diagnosis of BD. Additionally, at least two of the following must be present: genital ulceration, eye lesions, skin lesions, and positive pathergy test results. These criteria have demonstrated 95% sensitivity and 98% specificity. However, a key limitation is their exclusion of significant organ involvement, such as that reflected by vascular, neurological, and gastrointestinal complications [13].

In the ICBD scoring system, two points are assigned for each of oral ulcers, genital ulcers, and ocular lesions, and one point is assigned for each of positive pathergy test results and neurological and vascular involvement. Scores ≥4 lead to the diagnosis of BD [6]. In a multinational collaborative study involving patients in 27 countries, the ICBD demonstrated 94.8% unbiased sensitivity, markedly higher than that of the ISG criteria (85.0%), but with less specificity (90.5%) than the ISG criteria (96%) [6]. Particularly in the early stages of the disease, ICBD application may lead to overdiagnosis, with the misclassification of individuals with spondyloarthropathic features as having BD [6].

In 2016, an international expert consensus group introduced classification criteria specifically for pediatric BD [14]. The diagnosis of BD according to these criteria requires a minimum of three findings from distinct categories of oral, genital, skin, ocular, neurological, and vascular involvement [13]. For the pediatric population, these international criteria exhibit greater sensitivity (91.7%) but less specificity (42.9%) than the ISG criteria [15].

The primary objectives of BD management are to address the inflammatory flare-ups characteristic of the disease and prevent irreversible organ damage [16]. Such management necessitates consideration of various factors, including the type and extent of organ involvement, and patients’ demographic characteristics and treatment preferences. Given the diverse systemic manifestations of BD, an effective treatment approach mandates the implementation of a multidisciplinary strategy [1].

The use of topical corticosteroids, such as triamcinolone acetonide cream, is preferred for the initial treatment of isolated oral aphthae and genital ulcers [3]. Alternatively, topical sucralfate can be used alone or in combination with corticosteroids for these ulcers. Additionally, topical corticosteroids have proven effective in the management of anterior uveitis [17]. Colchicine is the preferred initial treatment for the prevention of recurrent mucocutaneous lesions in BD [1].

Systemic corticosteroids, whether administered orally as prednisolone or intravenously as methylprednisolone pulses, are commonly employed alone or with immunosuppressant drugs to manage various manifestations of BD [7]. They are particularly favored for the treatment of oral aphthae and genital ulcers that are unresponsive to topical therapy, and dermal lesions resistant to colchicine treatment [16].

## Case presentation

An eight-year-old Syrian girl presented to our Oral Medicine Clinic complaining of a recurrent deep painful oral ulcer. Her parents stated that she had been taking amoxicillin, clavulanic acid, acyclovir cream, and hyaluronic acid gel for 20 days. She had a history of gastrointestinal problems, including chronic constipation, as well as severe joint pain, and recurrent mouth ulcers since birth.

**Figure 1:**
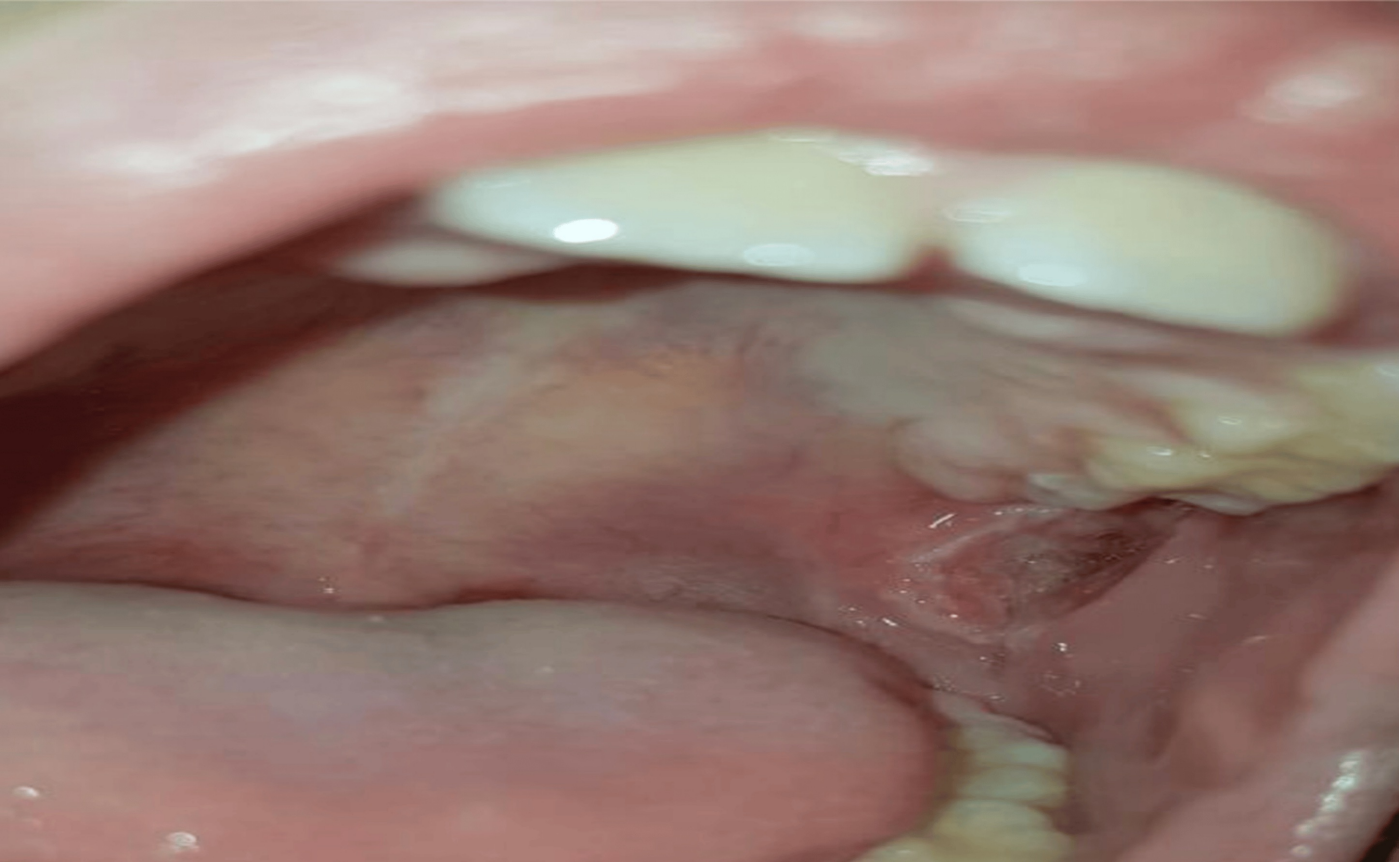
Deep ulcer located in the upper left mucobuccal fold, posterior to tooth #26.

Extraoral examination revealed tenderness of the bilateral submandibular lymph nodes. Intraoral examination revealed ulcer scarring on the left lateral border of the tongue, and a deep ulcer measuring approximately 2.0 × 1.0 cm in the upper left mucobuccal fold posterior to tooth #26 (Figure 1).

The patient was diagnosed with a recurrent major aphthous ulcer and prescribed Predo pediatric syrup as a mouthwash (1:1 dilution with lukewarm water, 2 min gentle gargling three times per day, no food or drink for five minutes thereafter) and paracetamol (500 mg as needed). She was referred to a gastrointestinal consultant for the evaluation of her chronic constipation.

At a one-week follow-up visit, an examination revealed that the ulcer had not responded to the medication, but that no new ulceration had developed. An excisional biopsy of the ulcer was performed under general anesthesia to rule out malignancy, as indicated by the patient’s physician. The histopathological report indicated that the excised tissue was benign squamous epithelium with reactive changes and surface erosion (healed ulcer). The patient’s physician prescribed colchicine 0.5 mg and indicated that the case be treated as BD.

Blood samples were collected from the patient; the HLA B-51 test was not available in the country, and it was sent to a laboratory outside the country to be tested. Laboratory results showed HLA B*51 positivity and antinuclear antibody, anti-myeloperoxidase immunoglobulin (Ig) G (ANCA), and anti-proteinase IgG (ANCA) negativity. The IgA, IgG, and IgM levels were normal, the CRP level was elevated (3. 8 mg/dL), and the rheumatoid factor level was decreased (<10 IU/mL). Complete blood counts were within the normal range, except for the elevation of neutrophils (63%). Renal profile results were within the normal range (Table 1).

**Table 1:** Laboratory investigations IgA: immunoglobulin A; mg/dL: milligrams per deciliter; IgG: immunoglobulin G; IgM: immunoglobulin M; ANCA: anti-neutrophil cytoplasmic antibody; HLA B-51: human leukocytes antigens-B51; mm/hr: millimeters per hour; IU/mL: international units per milliliter; ng/mL: nanograms per milliliter; WBC: white blood cells; RBC: red blood cells; L: liter; g/dL: grams per deciliter; MCV: mean corpuscular volume; fL: femtoliters; MCH: mean corpuscular hemoglobin; pg/cell: picograms per cell; MCHC: mean corpuscular hemoglobin concentration; RDW: red cell distribution width; CRP: C-reactive protein; mEq/L: milliequivalents per liter; mmol/L: millimoles per liter

Laboratory Test	Value (Reference Range)
IgA	106 mg/dL (33-236)
IgG	886 mg/dL (608-1572)
IgM	90 mg/dL (43-207)
Antinuclear Antibody	Negative
Anti Myeloperoxidase IgG (ANCA)	Negative
Anti Proteinase IgG (ANCA)	Negative
HLA B-51	Positive
Calcium	9.8 mg/dL (8.8-10.8)
Erythrocyte Sedimentation Rate	20 mm/hr (0-20)
Rheumatoid Factor	<10 IU/mL (10-14)
Vitamin D, 25 - Hydroxy	20.89 ng/mL (30.00-75.00)
WBC	9.07×109/L (5.00-15.00)
Neutrophils	63% (32.00-54.00)
Monocytes	6.7% (3.00-8.00)
Lymphocytes	29.4% (28.00-48.00)
Eosinophils	0.7% (0.00-3.00)
Basophils	0.2% (0.00-2.00)
RBC	4.88×1012/L (4.00-5.20)
Hemoglobin	12.1 g/dL (11.50-15.50)
Hematocrit	38.4% (35.00-45.00)
MCV	78.7 fL (77.00-95.00)
MCH	24.8 pg/cell (25.00-33.00)
MCHC	31.5 g/dL (32.00-36.00)
Platelets	293 ×109/L (150-450)
RDW	14.3% (11.50-15.00)
CRP	3.8 mg/dL (0.5-1.0)
Random Blood Sugar	83 mg/dL (70-140)
Sodium	139 mEq/L (135-145)
Potassium	4.97 mmol/L (3.50-5.10)
Chloride	105 mEq/L (98-107)
Bicarbonate	24 mEq/L (22-29)
Creatinine	0.49 mg/dL (0.33-0.70)
Urea Nitrogen	8 mg/dL (7-18)

**Figure 2:**
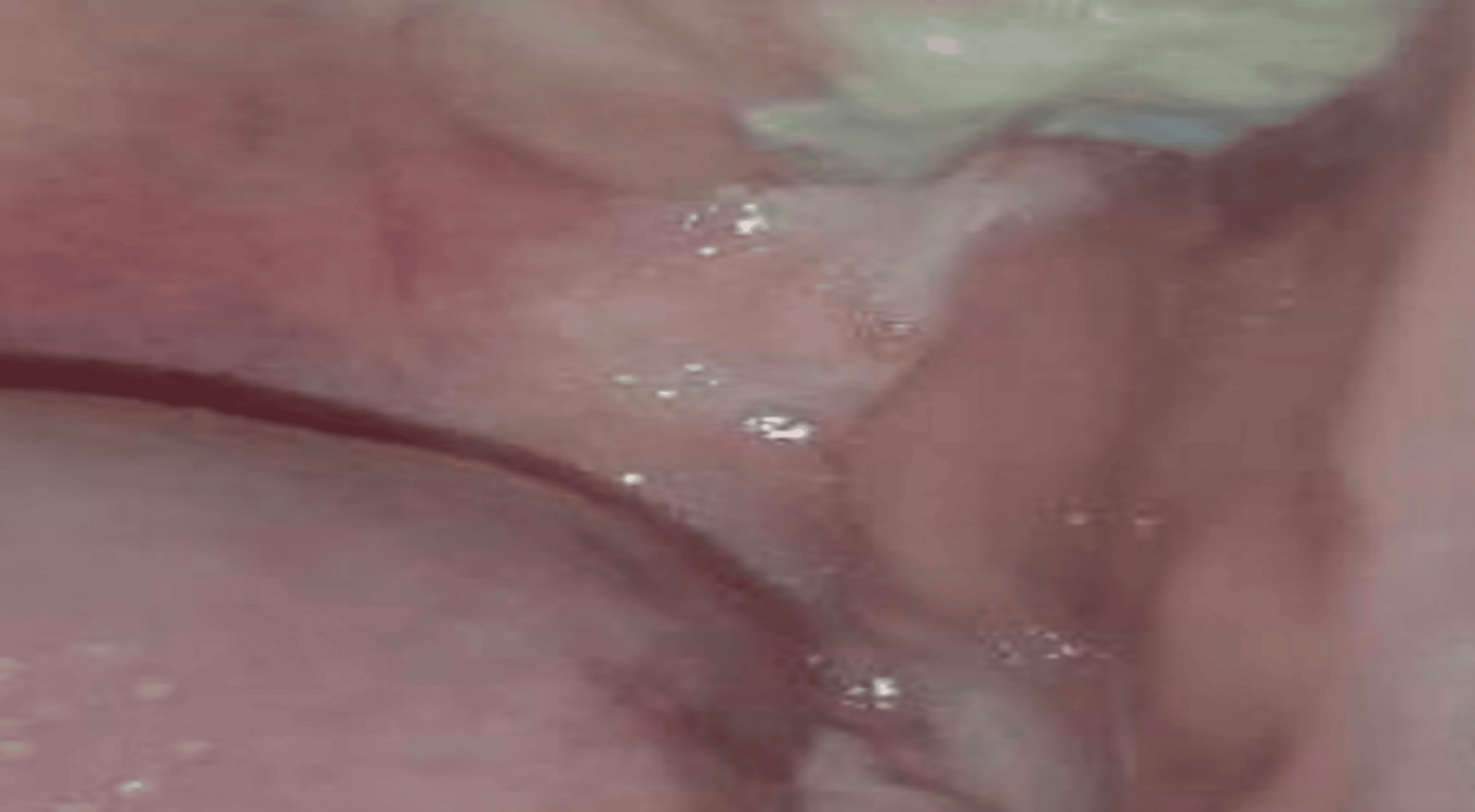
Scar in the upper left mucobuccal fold, posterior to tooth #26, following healing.

A follow-up examination performed after three months of colchicine treatment showed complete healing of the ulcer, with scarring (Figure 2).

One year after diagnosis and treatment with colchicine (initially 0.5 mg, later increased to 1.0 mg), the patient experienced complete resolution of recurrent oral ulcers. However, she developed side effects, notably myasthenia affecting the neck muscles, leading to discontinuation of the medication by her physician. Her parents have noticed and identified the triggers, such as ketchup, lollipops, and snacks with artificial flavors, and stopped them, which has improved her symptoms.

## Discussion

The symptoms of BD can vary widely, reflecting the different ways the disease can affect the body's blood vessels and tissues. The most common symptoms are mouth and genital ulcers, which often appear before other complications develop [7]. Considering the unique clinical presentation and disease course in children, the use of the pediatric-specific BD criteria is recommended for accurate diagnosis and management of this complex condition in the younger patient population. Genetic testing for the HLA-B51/B5 gene marker is an important diagnostic tool, as it is strongly linked to BD [10].

The established diagnostic criteria for pediatric BD these criteria require a minimum of three findings from distinct categories of mucocutaneous, ocular, neurological, and vascular involvement [13]. BD usually affects young adults (aged 20-40 years) and is seen less frequently in children [4]. The usual age at BD onset is about 30 years, but numerous observations and case series of pediatric cases have been reported [4,18]. The prevalence of BD in children is largely unknown because our thorough search for relevant literature and case studies uncovered only a small number of documented cases of BD in children. The extraordinary rarity of the pediatric case reported here stands out, as there appears to be limited published data on the occurrence of this condition in the young patient population.

The rarity of Behçet's syndrome in the pediatric population may confer certain advantages. Yazici et al. [12] noted that the lower incidence of cases is likely associated with a reduced overall healthcare burden and resource utilization relative to more common childhood conditions. Additionally, the specialized nature of BD may enable affected children to receive personalized care from clinicians with deep expertise in the management of the disease [1].

Conversely, the rarity of BD in children can present significant challenges. Koné-Paut [19] noted that practitioners’ unfamiliarity with the condition may lead to delays in accurate diagnosis, treatment, and management. Additionally, Davatchi et al. [6] noted the paucity of research, clinical data, and evidence-based guidelines for pediatric BD relative to adult BD. Finally, the low prevalence of the disease in pediatric patients may limit funding, resources, and incentives for the development of specialized treatments for this patient group [1].

In a prospective observational cohort study by Kone-Paut et al. examining 230 pediatric Behçet's cases, the median age of onset was 11 years [14]. Consistent with the review findings, oral aphthosis was a key feature in the female case, as it is reported in pediatric Behçet's cases. However, other classic Behçet manifestations like genital ulcers, ocular involvement, and skin lesions were absent in the current case, in contrast to the high rates noted in the study [14].

Similarly, a study by Koné-Paut et al. on 86 cases of pediatric Behçet's patients found much higher frequencies of mucocutaneous features compared to the present case [20]. The current patient's predominant symptoms of oral ulcers, joint pain, and gastrointestinal issues represent a more limited spectrum of Behçet's presentation. This aligns with the authors' observation that pediatric Behçet's can have a more severe disease course compared to adult-onset cases.

Interestingly, the current patient demonstrated HLA-B51 positivity, which is a common genetic association seen in pediatric BD cases [14]. This genetic marker, along with the patient's response to colchicine therapy, provides supportive evidence for Behçet's diagnosis in the current child.

Other conducted by Karincaoglu et al. found a higher frequency of neurological and gastrointestinal involvement in juvenile-onset cases, which was not observed in the presented patient. Additionally, the study revealed a higher incidence of genital ulcers as an onset manifestation in adult-onset BD, which was not a feature in this pediatric case [4].

## Conclusions

This case report highlights the unique and sometimes atypical presentation of Behçet's syndrome in the pediatric population. While sharing some similarities with previously published pediatric cases, such as young age of onset and oral ulcers, the current case exhibits a more limited spectrum of clinical features. This underscores the heterogeneity in pediatric Behçet's and the importance of maintaining a high index of suspicion, even when the full constellation of Behçet's symptoms is not present. Also, this study highlights that although recurrent, deep, and painful oral ulceration is a hallmark symptom of BD, the disease is quite rare in the pediatric population relative to the adult population. It provides the epidemiological context for the typically more common presentation of BD in adults than in young children, such as the eight-year-old female patient described here.

In our case, the trigger was the type of food as stated by the parents, snacks with artificial flavors trigger the initiation of the child’s symptoms as oral ulcers and chronic constipation. Avoiding such snacks leads to improvement of the condition.
